# X-Rays in the Treatment of Diseases of the Skin

**Published:** 1908-10-10

**Authors:** H. G. Adamson

**Affiliations:** Chief Assistant in the Skin Department at St. Bartholomew's Hospital; Physician to the Skin Department at Paddington Green Children's Hospital


### Hospital Clinics.

### X-RAYS IN THE TREATMENT OF DISEASES OF THE SKIN.

By H. G. ADAMSON, M.D., M.R.C.P., Chief Assistant in the Skin Department at St. Bartholomew's Hospital; Physician to the Skin Department at Paddington Green Children's Hospital.

X-rays are now so largely employed in the treatment of disease—and more especially in the treatment of skin affections—that the practitioner, although he may not be in a position to use this method himself, ought, at any rate, to understand its principles, and to know in what class of case it may be recommended. In the early days of the employment of *x*-rays in medicine there was hardly a skin complaint which was not tentatively subjected to this treatment.

The limits of its application are now becoming better known, and, while it has been found that the *x*-ray treatment is unsuited for many affections, in others its usefulness is undoubted. Of recent years the means of measuring the output of *x*-rays have been so far perfected that we are able to dose with precision and without fear of doing harm.

### Former Methods.

Formerly it was the custom to apply the rays without any accurate measurement of dose, and to give repeated short sittings under a tube emitting a comparatively feeble amount of *x*-rays. These applications were continued until some improvement occurred, or until a mild inflammatory reaction took place. But by this method there was always a danger of giving an overdose, for the effect of any one application of the rays does not appear until ten days or a fortnight afterwards, and consequently, since we continued to repeat our small doses right up to the time when a reaction was noticed, we had then already given more than was necessary. Now we are in a position to administer as a single dose exactly that amount of rays which can be absorbed by the skin without any damage to the normal tissues.

We have to remember that the effects of such a single full dose will not be felt until ten to fourteen days after the dose has been administered, and that it will continue for several days longer; so that, if it is necessary to give a second or more doses to the same area, we must wait at least three weeks before we do so. It follows that in place of a long series of more or less feeble doses administered three or four times a week, we now give a single full measured dose once in three or four weeks, with equal efficacy and with far greater safety.

### Modern Principles of Treatment.

I have already said that it was formerly thought necessary to produce an inflammatory reaction. Now we generally try to avoid this, because it is known that inflammatory reaction, according to its degree, is followed by more or less permanent damage to the normal skin. The principle upon which we act at the present time is the direct destruction of the diseased structures without damage to healthy tissues.

It is known nowadays that a sufficient dose of *x*-rays is destructive of living cells, and—a point which is of great importance—that the less stable the cell the more readily it succumbs; or, in other words, the cells of disease are destroyed by a somewhat smaller dose than are the more stable cells of the normal healthy tissue around it. By an accurate measurement of dose we are able to give to an affected part just sufficient *x*-rays to destroy the cells of disease without harming the healthy cells.

Before considering the particular forms of disease in which *x*-ray treatment is used it will be well to describe briefly the means employed for protecting the operator and for localising the effect of the rays to the diseased area, and also the method of ensuring accurate dosage.

### Method of Application and Dosage.

For the protection of the operator the *x*-ray tube is enclosed in a shield made of some material which is impervious to the rays. Those in common use are made either of lead-glass or of wood in the form of a box lined with a composition containing metal such as the protective aprons and gloves are made of. At one side of this shield there is an aperture through which the rays pass towards the part to be treated. On to this aperture are fixed cylindrical localisers, made of lead-glass or of metal, and of such length that when the affected part is applied to their free end it is at a fixed distance from the source of the rays—*i.e.* from the centre of the tube. The most convenient distance is found to be seven inches.

Mid-way between the source of the rays and the part exposed is fixed a holder, to which is attached during the exposure a pastille made of dried emulsion of platino-cyanide of barium spread on cardboard. This pastille is of a bright green colour, and it changes to a yellowish-brown under the influence of the rays. When, during an exposure, the pastille has changed to a standard yellow tint, we know that the right dose has been given—*i.e.* the fullest dose which the skin will stand without subsequent damage. This we call a Sabouraud pastille dose, from the name of the inventor of the pastille.

The time taken by the pastille to reach the standard tint will depend upon the quality and quantity of the rays emitted by the tube; or, to put it in another way, upon the vacuum of the tube and the amount of current passed through it. It is found convenient to work with tubes as far as possible of a certain vacuum, and to pass a certain current through them. With a tube giving an alternate spark of three inches and a current of one milliampere the time occupied in giving the dose is about fourteen minutes. The dose could be given in a shorter time by using a "harder" tube or by increasing the current, but there is then a smaller margin of safety in case we accidentally prolong the sitting.

### Cases Suitable for *x*-Ray Treatment.

We will now consider more particularly the sort of cases which are suitable for *x*-ray treatment. These may be divided into three groups:—

I. Those in which the lesions are made up of a new growth of fixed cells.

II. Chronic inflammations.

III. Cases in which we desire to modify some normal function—hair growth, sweat secretion, sebaceous secretion, nervous function.

The first group includes rodent ulcer and ordinary warts. Each of these affections consists in a new growth of epithelial cells, which, although of the same nature as the normal epidermis from which they have sprung, are yet of a less stable structure. It is found that by exposing these growths to the action of the rays we can destroy the unstable cells of the new growth without destroying those of the normal tissue around. To do this we must accurately measure the dose in the way I have already indicated.

### Warts and Rodent Ulcer.

To take common warts first: in most cases these may be destroyed effectually by local caustics; but where there are large numbers grouped together, a much more rapid and convenient way is to subject the area to the influence of *x*-rays. A single full pastille dose is given, and in most instances the warts on the area exposed shrivel up and disappear, without leaving any trace, in the course of two or three weeks. If we give less than a full dose they will subside for a time and then grow more luxuriantly; so the dose must be an accurate one. In a few cases a second dose (after an interval of not less than one month) may be required.

In the case of rodent ulcer we proceed in the same manner, beginning with a full pastille dose. The effect on a small ulcer will be to heal up the ulceration under a scab and to diminish the amount of growth in the raised "rolled edge" of the lesion. Usually, for a small ulcer, from six to ten sittings, at intervals of three weeks, are sufficient to cure the disease completely. In larger ulcers, and especially those with much new growth, many more sittings may be required, and it may also be necessary to pare off first of all some of the growth with the scalpel.

In these cases, too, better results are obtained by giving the pastille doses at intervals of two weeks instead of three or four. Some dermatitis with subsequent *x*-ray scar is produced, but this, I think, is justifiable in the case of an obstinate growth of this malignant nature. When a large ulcer is situated so that all its parts can be reached by the rays, when it does not already involve the deeper structures or is not accompanied by a large amount of new growth, it is generally possible to remove the disease completely and permanently, provided one perseveres with the treatment until no trace of the new growth remains.

*X*-ray applications have been suggested for the removal of nævi and moles. But for these cases the treatment is not suitable. The structures which make up these growths are just as stable as those of the surrounding tissues, and it is not possible to destroy them without also destroying the normal skin—*i.e.* without producing an *x*-ray burn—and replacing them by an *x*-ray scar, which is liable to give trouble in future owing to its tendency to become vascular and to break down and ulcerate.

### Chronic Inflammations.

Into the second group of cases we may put all those affections of the skin in which lesions are the result of chronic inflammatory reaction. Here we have a long list of diseases in which the *x*-rays may be applied, often with strikingly good results. The list includes acne vulgaris, acne rosacea, certain forms of lupus vulgaris, sycosis menti, localised areas of chronic eczema, of lichen planus, or of psoriasis.

The manner in which the rays act upon chronic inflammatory lesions is not altogether clear. It seems probable that they directly destroy the inflammatory cells just as they do those of the new growth, and that these, being less stable than the cells of the normal tissue, are affected first.

The most favourable cases in which to watch the effect of *x*-ray applications on chronic inflammations are those forms of lupus which are met with about the hands—namely lupus verrucosus or verruca necrogenica. Here we have a chronic inflammation in which overgrowth of the epidermal cells plays a prominent part. Under the action of the rays these lesions quickly dry up and finally disappear entirely.

In cases of acne vulgaris, with greasy skin, comedones and papulo-pustules, a series of *x*-ray applications (always in measured doses at fixed intervals) causes cessation of the oily secretion, shrivelling up of the horny comedo plugs, and disappearance of the inflammatory papules. In acne rosacea with inflammatory nodules these latter rapidly dwindle away, and even the erythematous condition subsides.

### The *X*-Rays in Active Inflammatory Conditions.

Supposing, however, we are treating a case of pustular sycosis of the beard region, or a case of acne vulgaris with much pustulation, then we have to remember that events do not always run so smoothly. For if the inflammation is at all active the immediate effect of the rays is to increase the activity, and there is often an alarming increase of pustulation and diffuse inflammation. Possibly what happens is the liberation of still virulent micro-organisms by the death of the phagocytic cells, and so a renewed effort of response on the part of the tissues.

This alarming increase of inflammation is, however, temporary, and it subsides after a while, leaving the general condition of the parts much improved. It is not to be confused with a reaction due to overdose of *x*-rays, and does not lead to subsequent damage of the normal structures.

In almost any chronic inflammatory dermatitis of limited extent—chronic eczema, lichen planus, psoriasis, for example—*x*-ray applications may be employed with excellent result. Here it is as well to give only half to three-quarters of a pastille dose at each sitting.

### Lupus Vulgaris.

While discussing the treatment of chronic inflammations by $x$-rays, a few remarks may be made in reference to the treatment of lupus vulgaris. I have already stated that lupus verrucosus does extremely well. In ordinary lupus vulgaris, with apple-jelly nodules, $x$-ray applications have little or no effect; we cannot produce resolution of the nodules by means of pastille doses—that is to say, by doses which do not lead to $x$-ray dermatitis; and the method (sometimes employed) of actually burning out the nodules by production of an $x$-ray ulceration is not good treatment. $X$-ray applications, however, are very useful indeed as a preliminary treatment where there is much crusting or ulceration: the crusts disappear and the ulcerations heal, leaving then only the typical lupus nodules, which can be dealt with by other means, notably by Finsen light treatment. Recently a method has been employed with success of first of all ulcerating the lupus nodules by salicylic acid plasters, or of scraping them with a sharp spoon, and then healing up the ulceration with $x$-ray applications.

### Modification of Skin Functions by $x$-Rays.

In the third group of cases we aim, not at the destruction of some morbid growth, but at the modification of one or other of the physiological functions of the skin—that is to say, of the sebaceous gland secretion, of the sweat production, of the growth of hair, or of the nervous mechanism. It appears that the cells of the sweat glands, of the sebaceous glands, of the hair bulbs and papillæ, and of the nerve terminals are more sensitive to the action of the $x$-rays than are the cells of the derma and of the epidermis; for it is found that by giving first the right dose of $x$-rays we can influence these cells without damaging the surrounding tissues. For example, we can destroy the delicate cells of the hair bulb, and so cause fall of hair, without damaging the skin; and we can do this without permanent loss of hair if our dose is so adjusted that it does not totally destroy the hair follicle or the basal cells of the epidermis from which the new hair follicle is subsequently regenerated.

### Depilation in Ringworm.

We make use of this depilatory action of the $x$-rays in the treatment of ringworm of the scalp and of ringworm of the beard. The well-known difficulty of curing these cases is due to the fact that the ringworm fungus invades the hair as far as its root, and that consequently we are unable to reach it by local parasiticides. By means of the $x$-rays we are able to cause the hair to fall, and with it, of course, the fungus. The rays do not kill the fungus, but simply remove the hair upon which it feeds.

The operation is a very delicate one, for the dose must be carefully adjusted, or permanent damage may be done to the hair follicles and epidermis, and no regrowth of hair will take place. In order to cure a patch of ringworm, the affected area is placed at a fixed distance from the source of the rays (*i.e.* the centre of the tube), and a single dose (measured by the pastille of Sabouraud), is given. In two weeks from this time the hair will begin to fall, and in three weeks the area exposed will be completely bald of healthy as well as infected hairs. The new hair will begin to grow in another six weeks or two months.

The action of the rays upon the cells of the sweat glands and of the sebaceous glands is made use of in the treatment of hyperidrosis and of oily seborrhœa. Sweating of the hands or of the axillæ may be cured by exposure to the $x$-rays. Here also it is necessary to give measured doses, and a full pastille dose is given, followed, if necessary, by another at an interval of not less than one month. The results are generally very satisfactory, and apparently permanent.

$X$-ray applications are also of very great value in the treatment of localised pruritus, especially pruritus ani and pruritus vulvæ. They apparently act by a direct influence upon the cells of the terminal nerve filaments. At any rate, after one or several rather less than full pastille doses the pruritus generally ceases, and the cure is often permanent.

To recapitulate briefly: (1) the $x$-rays are useful mainly in the following commoner skin affections:—Rodent ulcer, warts, acne vulgaris, acne rosacea, sycosis menti, ringworm of the scalp or beard, hyperidrosis, pruritus ani or vulvæ, lupus verrucosus, and lupus vulgaris, as a preliminary treatment for ulcerated or crusted lesions, or after scraping; (2) it is of the utmost importance always to administer the $x$-rays in accurately measured doses at measured intervals, and with these precautions (and only with these precautions), the method is an absolutely safe one, and forms a very valuable addition to the therapeutics of skin affections.

